# Depletion of the RNA-Binding Protein RBP33 Results in Increased Expression of Silenced RNA Polymerase II Transcripts in *Trypanosoma brucei*

**DOI:** 10.1371/journal.pone.0107608

**Published:** 2014-09-12

**Authors:** Sandra M. Fernández-Moya, Mark Carrington, Antonio M. Estévez

**Affiliations:** 1 Instituto de Parasitología y Biomedicina “López-Neyra”, IPBLN-CSIC, Armilla, Granada, Spain; 2 Department of Biochemistry, University of Cambridge, Cambridge, United Kingdom; Keio University, Japan

## Abstract

We have characterized the RNA-binding protein RBP33 in *Trypanosoma brucei*, and found that it localizes to the nucleus and is essential for viability. The subset of RNAs bound to RBP33 was determined by immunoprecipitation of ribonucleoprotein complexes followed by deep sequencing. Most RBP33-bound transcripts are predicted to be non-coding. Among these, over one-third are located close to the end of transcriptional units (TUs) or have an antisense orientation within a TU. Depletion of RBP33 resulted in an increase in the level of RNAs derived from regions that are normally silenced, such as strand-switch regions, retroposon and repeat sequences. Our work provides the first example of an RNA-binding protein involved in the regulation of gene silencing in trypanosomes.

## Introduction

Trypanosomatids are single-celled eukaryotes that are responsible for major human and livestock diseases. Genome organization in these organisms is unusual for a eukaryote, as open reading frames are organized in long polycistronic transcription units (TUs) that are transcribed by RNA polymerase II from only a few initiation sites in the chromosome [1]. Individual mRNAs are co-transcriptionally processed from polycistronic precursors by coupled *trans*-splicing at the 5′-end and polyadenylation at the 3′-end [2]. Adjacent TUs are often convergent or divergent, and are separated by strand switch regions (SSRs). RNA polymerase II transcription usually begins at divergent SSRs and ends at convergent SSRs [1], [3]. Histone variants present in convergent SSRs are distinct from other variants associated with divergent SSRs, and it is assumed that the limits of polycistronic TUs are defined by distinct chromatin conformations [4]. Therefore, RNA polymerase II-dependent transcription initiation and termination seems to be regulated simply by histone modifications and chromatin structure, but the mechanism is currently unknown. In addition, chromatin conformation could be important in insulating RNA polymerase II TUs from RNA polymerase I and III expression sites [4], .

There are numerous repetitive sequences within the *Trypanosoma brucei* genome, including retroposons INGI/RIME and SLACs, and satellite-like repeats such as CIR147 [6]. Transcripts derived from these elements are selectively degraded by the RNA interference (RNAi) pathway [6]. It is currently unknown whether chromatin structure also plays a role in the transcriptional silencing of retroposons and repeats. That could well be the case, since some histone variants that are known to be involved in heterochromatin-dependent silencing in other organisms, such as H2AZ, are associated with repetitive DNA in *T. brucei* [7].

In other eukaryotes, non-coding RNAs (ncRNAs) and RNA-binding proteins (RBPs) are emerging as important factors regulating gene silencing and chromatin structure through RNAi-dependent and independent mechanisms. Examples include RNAi-mediated formation of heterochromatin at non-coding and repeated DNA in *Saccharomyces pombe* [8], or transcriptional silencing promoted by the association of chromatin-remodeling complexes with ncRNA-binding proteins such as IDN2 in *Arabidospsis thaliana* [9] or Nrd1 in *S. pombe* and *S. cerevisiae* [10]. Whether a similar scenario also occurs in trypanosomes is not known at present.

In this work we describe the characterization of the RNA-binding protein RBP33, which was first identified in a search for proteins that were able to bind to a uridine-rich RNA element *in vitro* [11]. RBP33 depletion results in the accumulation of transcripts derived from silenced regions, such as SSRs and retroposon genes, and thus it could have a role in regulating gene silencing in trypanosomes.

## Results

### RBP33 is an essential nuclear protein in trypanosomes

**Figure 1:**
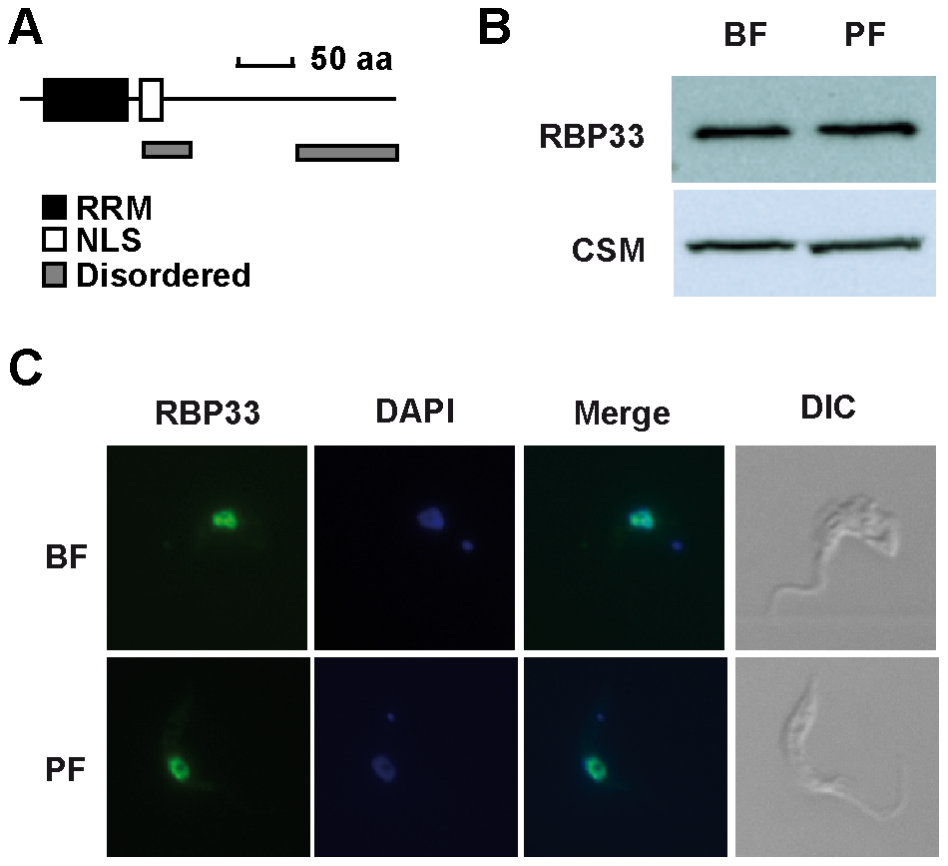
Expression and subcellular localization of RBP33 in bloodstream and procyclic trypanosomes. (A) Schematic diagram of *T. brucei* RBP33. An anti-RBP33 antiserum was used to detect the protein by (B) western blot analysis of total cell extracts or (C) immunofluorescence assays. CSM protein [39] was included as loading control, and DAPI was used to stain nuclei.

**Figure 2:**
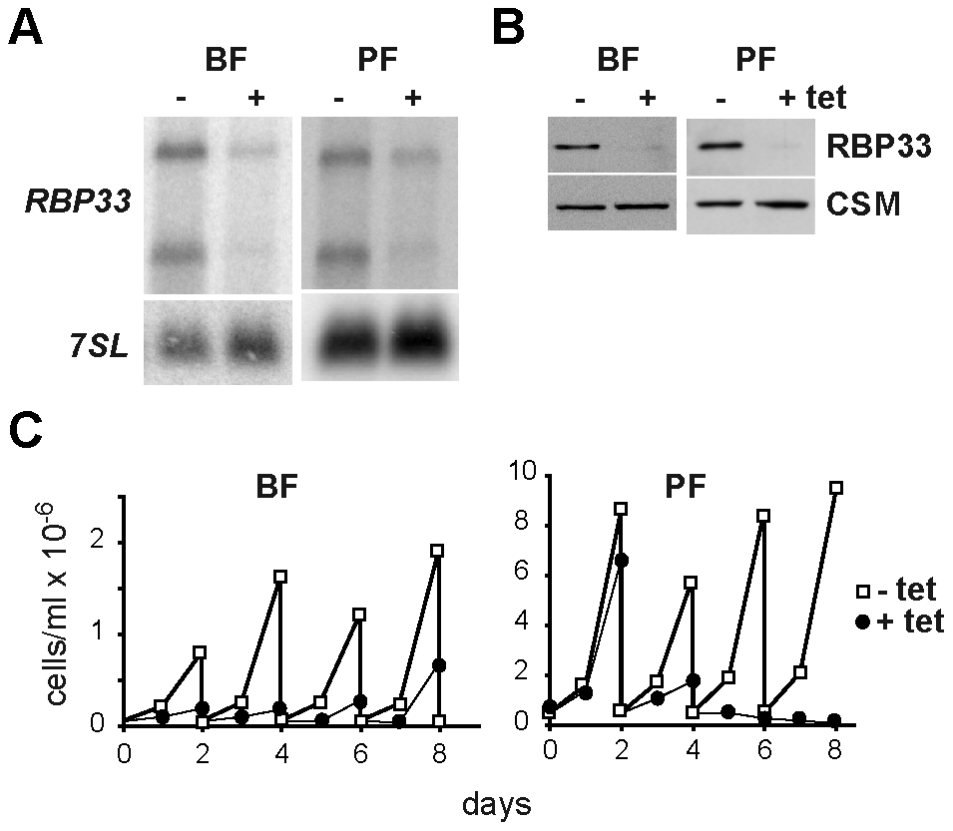
RBP33 is an essential protein in trypanosomes. Cell lines were generated that expressed *RBP33*-specific dsRNA in a tetracycline-inducible manner. The levels of mRNA (A) or protein (B) were analyzed in cells incubated with tetracycline for 48 h. Loading controls were the *7SL* RNA [40] or the CSM protein [39]. (C) RNAi cell lines were grown in the absence (open squares) or presence (filled circles) of 1 µg/ml tetracycline to induce RNAi. Cultures were followed up to 8 days and diluted to 0.5×10^5^ cells/ml (bloodstream) or 0.4×10^6^ cells/ml (procyclic) every 2 days as required.

The *T. brucei* RNA-binding protein RBP33 (Tb927.8.990) is 317 amino acids long, and contains an RNA-recognition motif, a putative nuclear localization signal and two intrinsically disordered regions (Fig. 1A). Its carboxyl half has no apparent similarity to any known protein outside the *Trypanosomatidae* family, and it is predicted to have intrinsically disordered regions also in other trypanosomatid species (Fig. S1). Western blot analysis of total cell extracts using a polyclonal antiserum raised against the carboxyl half of RBP33 [11] revealed a single band with an apparent electrophoretic mobility of 40 kDa, which was expressed at similar levels in the two readily cultured developmental forms, the mammalian bloodstream and the insect procyclic forms (Fig. 1B and Fig. S2A). RBP33 localized mainly in the nucleus, as judged by immunofluorescence assays (Fig. 1C). The abundance of RBP33 was estimated to be ∼50.000 molecules per cell, as judged by Western blot analysis with proyclic cell extracts and recombinant RBP33 (Fig. S2), which is within the range described for other RNA-binding proteins in trypanosomes (reviewed in [12]). RBP33 expression was silenced *in vivo* using RNA interference (RNAi) in a tetracycline-inducible fashion. Although RNAi was not very efficient at the mRNA level, a marked reduction in protein abundance was observed after 48 h of tetracycline induction (Fig. 2A–B and Fig. S2B). The presence of two bands in the Northern blot assays most likely represents transcripts with different 3′UTRs, as suggested by global transcriptome surveys [13]. Depletion of RBP33 resulted in cell death, indicating that this protein is essential in both the developmental forms tested (Fig. 2C).

### RBP33 binds to a specific cohort of RNAs

**Figure 3:**
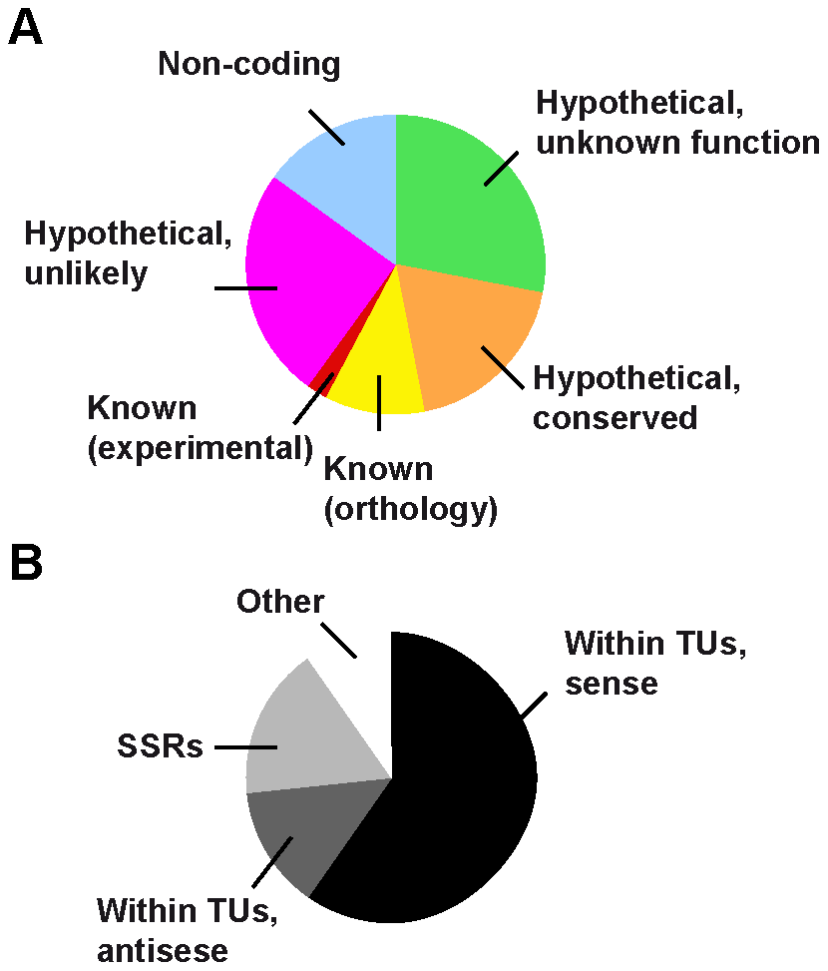
Distribution of RBP33-bound transcripts. (A) RNAs associated with RBP33 (log_2_FC>3) were categorized according to whether they are predicted to encode functional proteins. Color code is based on that of GeneDB (http://www.genedb.org/Homepage/Tbruceibrucei927). (B) Localization of the genes encoding RBP33 targets (log_2_FC>3) on the genome. ‘SSR’ refers to genes localized in the vicinity of strand-switch regions, whereas ‘Other’ indicates genes found close to the beginning or end of chromosomes, or close to snoRNA or pseudogene clusters.

**Figure 4:**
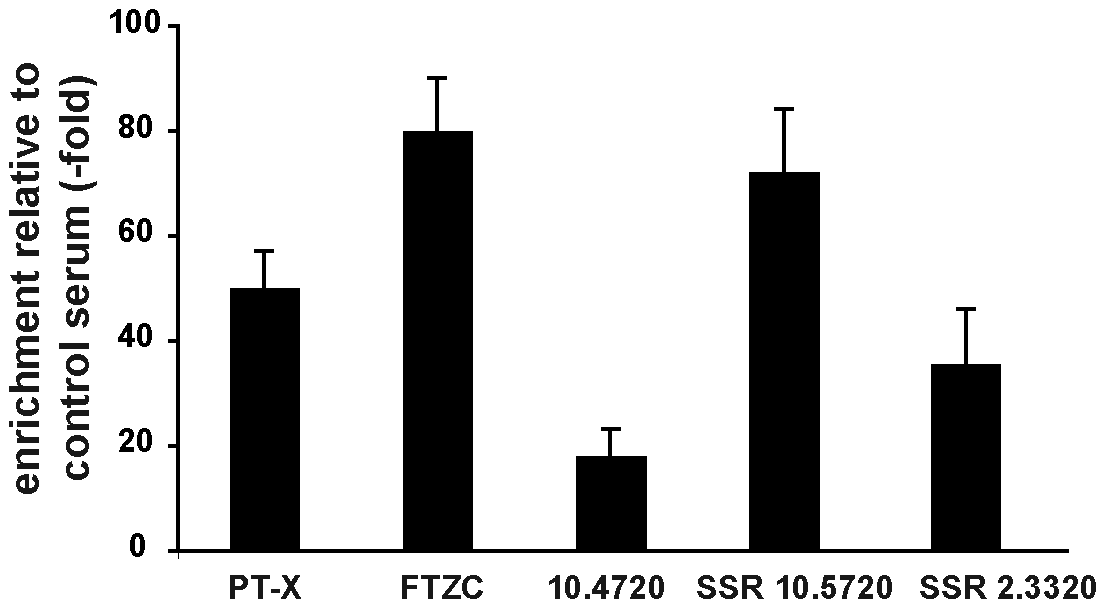
Validation of RBP33 targets. Quantitative RT-PCR assays were performed to confirm binding of three coding RNAs (pteridine transporter *PT-X*, Tb927.10.9080; flagellar transition zone component *FTZC*, Tb927.10.8590; and Tb927.10.4720) and transcripts derived from the SSR downstream gene Tb927.10.5720 or from the SSR downstream gene Tb927.2.3320.

To gain insight into RBP33 function, we sought to identify the set of RNAs associated to RBP33 using immunoprecipitation of RBP33 ribonucleoprotein complexes followed by RNA-seq deep sequencing. In the immunoprecipitated material, we could identify ∼430 transcripts that were enriched 4-fold or more (log_2_FC>2) with respect to input RNA, and ∼170 transcripts enriched 8-fold or more (log_2_FC>3) (Table S1). Examples of RNA-seq alignments visualized using the Artemis software can be seen in Fig. S3A. Only a minority of these enriched RNAs encoded *bona fide* proteins according to the annotated reference genome (http://www.geneDB.org/Homepage/Tbruceibrucei927) and available ribosome profiling data [14]. There was no obvious functional relationship between the proteins encoded by these mRNAs. Interestingly, RBP33 binds to its own mRNA (Table S1 and Fig. S3A). Most RBP33-associated RNAs are either non-coding or annotated as encoding ‘hypothetical proteins’ or ‘hypothetical proteins, unlikely’ (Fig. 3A), and are present at minimal levels within the cell, according to the low number of reads obtained in our input RNA sample (Table S1) and in other genome-wide analyses [13], [15]. When we analyzed the chromosome location of these genes, the majority (60%) were found within polycistronic units in sense orientation, whereas 17% localized to the vicinity of strand switch regions and 13% showed an antisense orientation within a transcription unit (Fig. 3B). We could confirm binding of RBP33 to transcripts derived from both protein-coding genes and convergent SSR regions by quantitative RT-PCR analysis of the immunoprecipitated material (Fig. 4). Although RBP33 could interact directly with RNA, we cannot rule out the possibility that RBP33 is in a complex with other proteins that directly bind to target transcripts.

### RBP33 silencing increases the levels of SSR-derived transcripts

**Figure 5:**
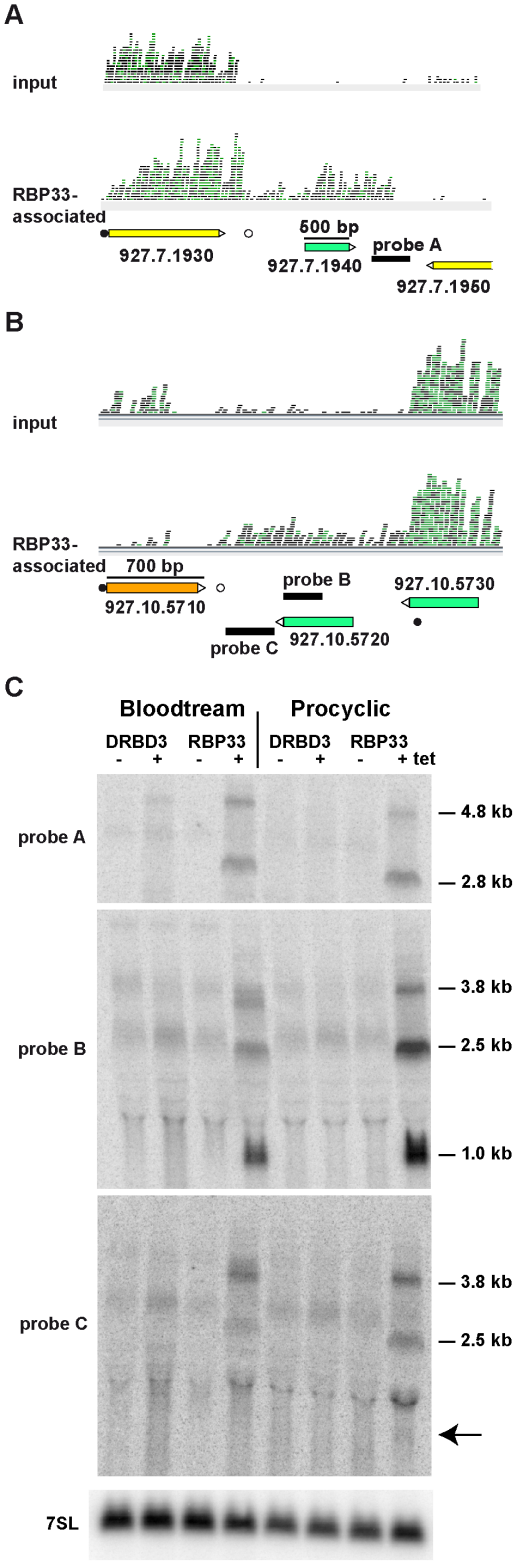
Effect of RBP33 depletion on the levels of SSR-derived transcripts. (A) Artmemis visualization of RNAseq data corresponding to the SSR downstream gene Tb927.7.1930 and to the SSR downstream gene Tb927.10.5710. Probes used for Northern blot analysis are indicated. ‘Input’ refers to the RNA-seq data obtained from total RNA before immunoprecipitation, whereas ‘RBP33-bound’ refers to the immunoprecipitated RNA using RBP33 specific antibodies. Annotated splice-acceptor and polyadenylation sites [13] are indicated as filled or empty circles, respectively. The splice-acceptor site for Tb927.10.5720 has been mapped in this study, and lies within the downstream open-reading frame. (B) Northern hybridizations using radiolabeled double-stranded DNA probes as indicated in panel A. An RNAi cell line expressing dsRNA against the essential RNA-binding protein DRBD3 [11] was used as a control. Total RNA was obtained from parasites grown in the absence (−) or the presence (+) of tetracycline for 48 h, transferred to nylon membranes and hybridized with specific probes. The *7SL* RNA was used as a loading control. The arrow indicates the position of the 1.0 kb transcript detected with probe B. Representative blots are shown.

We next analyzed the effect that RBP33 depletion had on the levels of two RBP33 targets encoding *bona fide* proteins, the pteridine transporter Tb927.10.9080 and the flagellum protein FTZC (Tb927.10.8590). Silencing of RBP33 did not have a major effect on the abundance of either transcript; FTZC protein levels were not altered either (Fig. S4). In contrast, the levels of several SSR-derived transcripts increased dramatically upon RBP33 silencing. As shown in Fig. 5A, a probe designed to detect transcripts derived from the convergent SSR between genes Tb927.7.1940 and Tb927.7.1950 (probe A), hybridized with two RNA species of 4.8 kb and 2.8 kb in both bloodstream and procyclic trypanosomes when RBP33 was ablated by RNAi. Accumulation of SSR-derived transcripts was not due to the slowing in growth and cell death associated with RBP33 RNAi as it was not observed upon depletion of DRBD3, an unrelated and essential RNA-binding protein [11]. In the case of the SSR between genes Tb927.10.5710 and Tb927.10. 5720 (Fig. 5C), a probe hybridizing to the latter gene (probe B) detected three RNA species of 3.8 kb, 2.5 kb and 1.0 kb, whereas a probe hybridizing upstream (probe C) only detected the two larger RNAs. The three transcripts detected with probe B are likely to be polyadenylated, since they decreased in size upon incubation with RNaseH in the presence of oligo(dT) (Figure S5). We could map the splice-acceptor site for transcript Tb927.10.5720 to a canonical AG dinucleotide located 396 nt upstream the initiation codon (Figure 5B).

### Retroposon- and repeat-derived transcripts also increase in abundance upon RBP33 depletion

**Figure 6:**
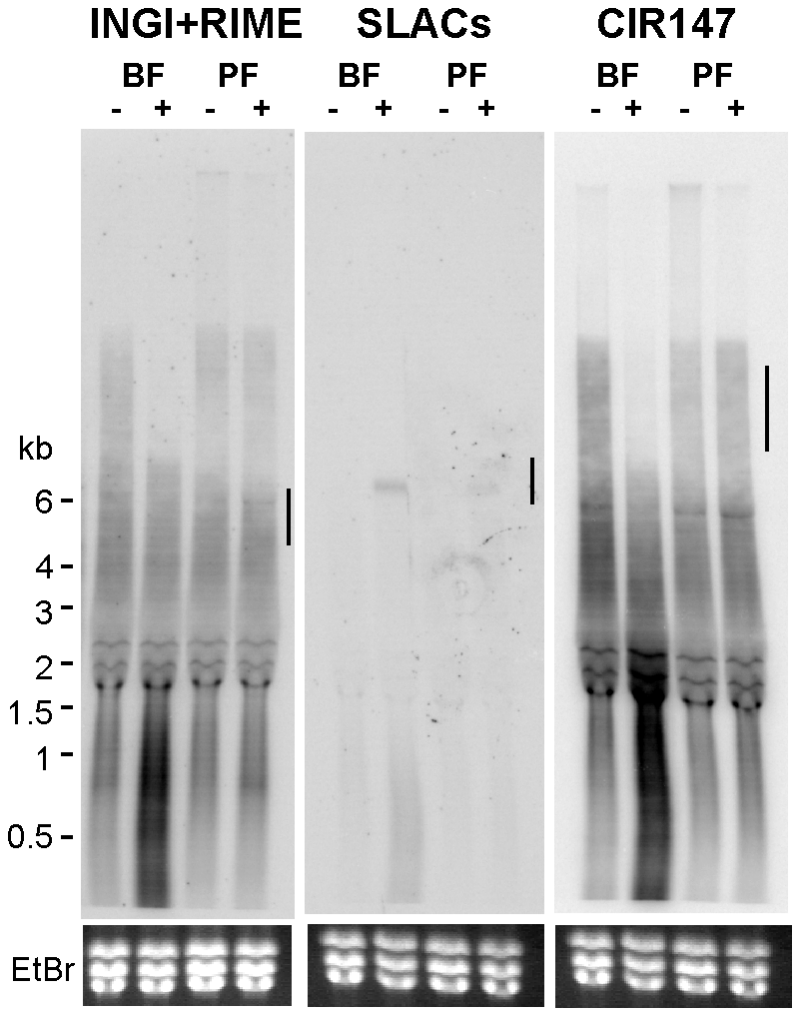
Effect of RBP33 depletion on the levels of transcripts derived from retroposons and CIR147 repeats. Probes designed to detect both INGI/RIME-derived transcripts, SLACs retroposons or CIR147-derived transcripts were used in Northern hybridizations as described in Fig. 5 legend. Ethidium bromide staining was used as a loading control. Vertical lines indicate the expected migration of the corresponding transcripts upon disruption of the RNAi machinery observed in [6].

Since convergent SSR regions are not transcribed in physiological conditions, we wondered whether RBP33 ablation could also promote the accumulation of other transcripts that are likewise silenced, such as retroposons INGI/RIME and SLACs, and CIR147 repeat transcripts [6]. Indeed, upon depletion of RBP33 there was an evident increase in the abundance of transcripts derived from both INGI and RIME retroposons, SLACs retroposons and CIR147 repeats (Fig. 6). A probe designed to detect only INGI-derived transcripts yielded similar results (data not shown). Interestingly, this phenomenon appears to be stage-specific, as the accumulation of transcripts was immediately apparent in bloodstream form cells but only marginal in procyclic form cells (Fig. 6).

## Discussion

In this work, we have undertaken the characterization of the RNA-binding protein RBP33. We found that the protein is localized in the nucleus, and is essential in both bloodstream and procyclic trypanosomes. Using RNA immunoprecipitation and deep sequencing, we were able to identify a cohort of transcripts associated with RBP33 and thus obtain valuable information about the function of this protein. Although RBP33 binds to mRNAs encoding known or conserved proteins, most of its RNA targets are likely to be non-coding and/or present at minimal levels in the cell. Indeed, 90% of the RBP33-associated RNAs annotated as coding for ‘hypothetical proteins’ or ‘hypothetical proteins, unlikely’ were not detected by ribosome profiling [14], and 75% were not detected in a global transcriptome survey [13] (Table S1). Moreover, over one-third of the corresponding genes are located next to SSRs, close to the ends of chromosomes or have an antisense orientation within a transcription unit (Fig. 3 and Table S1). Interestingly, depletion of RBP33 results in an accumulation of SSR-derived transcripts. In the related parasite *Leishmania tarentolae*, it has been shown that base J (ß-D-glucosyl-hydroxymethyluracil) prevents read-through at RNA polymerase II termination sites [16]. *L. tarentolae* cells seem to require base J for viability [17], but *T. brucei* can survive without J [18]. Two thymidine hydrolases are involved in the synthesis of J [18], but none is encoded by RBP33-bound mRNAs. To our knowledge, this is the first instance of a regulatory protein that has a role in silencing gene expression at RNA polymerase II termination regions. In addition, RBP33 silencing promotes the accumulation of retroposon and repeat-derived RNAs. This phenomenon has been also observed upon disruption of the RNAi machinery in *T. brucei* [6], raising the question of whether RBP33 could have a role in the regulation of the RNAi pathway in trypanosomes. In fact, convergent SSRs have been shown to produce small interfering RNAs (siRNAs) in *T. brucei* [19]. However, the molecular phenotype observed upon depletion of RBP33 presents some differences with that observed in trypanosomes lacking a functional RNAi pathway. Thus, deletion of the genes encoding the dicer-like protein TbDCL2 or the argonaute protein TbAGO1 results in the accumulation of full-length INGI transcripts which are ∼6 kb long and CIR147-derived transcripts of ∼10 kb [6], whereas the transcripts observed upon RBP33 silencing were smaller (Fig. 6). We did, however, detect full-length transcripts corresponding to SLACs retroposons. Second, the accumulation of retroposon and repeat transcripts is stage-dependent, being almost undetected in procyclic trypanosomes. And third, deletion of TbDCL2 did not result in the accumulation of convergent SSR-derived transcripts, whereas this sort of RNAs was readily visible upon RBP33 ablation. For instance, we could detect transcripts derived from the SSR located in between genes Tb927.10.2390 and Tb927.10.2400 (Figure S3B), whereas no accumulation of RNAs transcribed from this region was observed when TbDCL2 was deleted [19].

SSRs are enriched in specific histone variants in trypanosomes [4] but whether these variants promote heterochromatin assembly and thus transcription silencing is not known at present. Likewise, it is unknown whether the silent transcriptional status of CIR147 repeats and retroposon genes is due to heterochromatinization, although some histone variants that are known to be involved in heterochromatin-dependent silencing, such as H2AZ, have been found associated to repetitive DNA [7]. In other organisms, heterochromatin formation depends on both RNAi-dependent and RNAi-independent mechanisms. Among the latter, the yeast RNA-binding protein Nrd1 has been shown to bind to unstable non-coding RNAs (ncRNAs), and to promote heterochromatin assembly and transcriptional silencing, thus providing a failsafe termination mechanism in case of read through transcription [20], [21]. RBP33 could perform a similar function in trypanosomes by binding to non-coding RNAs and promoting gene silencing *via* chromatin conformation. The source of ncRNAs could be SSRs, genes whose products are annotated as ‘hypothetical proteins (unlikely)’ or even transcripts coding for known proteins. In this regard, we have shown that although RBP33 binds to translatable transcripts, its depletion has no apparent effect on the levels of target mRNAs or in the protein product. However, RBP33 could be involved in the processing of cryptic ncRNAs located within the untranslated regions of a defined set of mRNAs. Alternatively, RBP33 could be part of a surveillance mechanism that selectively degrades RNAs transcribed from silenced regions in the genome. Another plausible interpretation is that RBP33 depletion results in an increased maturation or alternative processing of transcripts derived from SSR and retroposons, which is supported by the observation that slower-migrating INGI/RIME and CIR147 repeats transcripts decrease in abundance upon RBP33 silencing in bloodstream forms (Fig. 6). The actual mechanism remains to be determined. Our findings support the hypothesis that gene silencing is, at least in part, controlled at the RNA level in trypanosomes, and uncovers an unprecedented role for RNA-binding proteins in this process.

## Materials and Methods

### Trypanosome culture and RNA interference

*Trypanosoma brucei* 449 procyclic cells [22] were grown at 27°C in SDM-79 medium [23] containing 10% fetal bovine serum. ‘Single marker’ *T. brucei* Lister 427 bloodstream cell line S16 [24] were maintained in HMI-9 medium [25] containing 10% fetal bovine serum at 37°C with 5% CO_2_. For RNAi studies, a stem-loop strategy was followed to generate dsRNA [11]. A fragment of the RBP33 ORF corresponding to the carboxyl half of the protein was PCR-amplified and cloned into pGR19 [26] to yield pGR70. Procyclic and bloodstream trypanosomes were transfected with pGR70 linearized with *Not* I and selected in the presence of 50 µg/ml (procyclic) or 5 µg/ml (bloodstream trypanosomes) hygromycin. For RNAi induction, tetracycline was added to the culture medium at a concentration of 1 µg/ml.

### Protein analysis

Polyclonal antibodies against the last 160 amino acids of *T. brucei* RBP33 were raised in rabbits and affinity-purified using a full-length version of the protein (described in [11]). Immunoblotting and immunofluorescence analysis were carried out as in [27], [28]. Protein motifs were analyzed using PSORT [29] and SMART [30]. Intrinsically disordered regions were identified using GlobPlot [31], IUPred [32] and PrDOS [33] software.

### RNA analysis

Northern hybridizations and quantitative RT-PCR analysis were done as described [11], [34]. The oligodeoxynucleotides used in this work are listed in Table S2.To map the splice-acceptor site for Tb927.10.5720 transcript, cDNA was obtained from RBP33-depleted RNA samples using oligodeoxynucleotide AE340, and conventional RT-PCR reactions were carried out using AE340 and a sense oligodeoxynucleotide corresponding to the *T. brucei* spliced leader sequence. To remove poly(A) tails, 20 µg of total RNA obtained from RBP33-depleted samples were incubated with 300 pmoles of oligo(dT)_20_, heated at 65°C for 10 min, transferred to a water bath at 42°C, cooled to 30°C, treated with 5 U of RNAseH (Invitrogen) for 30 min at 37°C and subjected to Northern hybrization. Samples lacking oligo(dT)_20_ were treated identically and analyzed in parallel.

To identify the cohort of transcripts bound to RBP33, 2×10^9^ procyclic trypanosomes were washed in ice-cold Voorheis's-modified phosphate-buffered saline (vPBS; PBS supplemented with 10 mM glucose and 46 mM sucrose), resuspended in 1 ml of vPBS and UV-irradiated twice at 400 mJ/cm^2^ in a Stratalinker apparatus (Stratagene). Cells were resuspended in 2 ml of 10 mM Tris-HCl, pH 7.4, 2 mM DTT, 1 mM EDTA, 0.1% Igepal, 50 U/ml RNaseOUT (Invitrogen) and protease inhibitors (complete mini EDTA-free cocktail, Roche) and lysed by passing the suspension through a 27-gauge syringe thrice on ice. The cell extract was centrifuged at 16,000×*g* for 10 min at 4°C, and NaCl was added to the supernatant at a final concentration of 150 mM. This final lysate was incubated with 2 µg of affinity-purified anti-RBP33 antibody for 3 h at 4°C. To capture RBP33 ribonucleoprotein particles, 150 µl of 50% protein A agarose slurry (Calbiochem) were added to the lysate. After an incubation of 1.5 h at 4°C, beads were transferred to a disponsable column (Biorad), washed twice with 10 ml of washing buffer (10 mM Tris-HCl, pH 7.6, 150 mM NaCl, 1 mM EDTA) and once with 2 ml of washing buffer containing 1 M urea, resuspended in 450 µl of 10 mM Tris-HCl, pH 8, 100 mM NaCl, 0.5% SDS, 1 mM EDTA and incubated in the presence of 100 µg of proteinase K (Ambion) for 30 min at 50°C. RBP33-associated RNAs were obtained by phenol:chloroform extraction and ethanol precipitation, and were DNaseI-treated prior to sequencing.

cDNA libraries for high-throughput sequencing were obtained from two biological replicates using the Illumina mRNA-seq kit according to manufacturer's instructions. Libraries were sequenced on an Illumina platform at EASIH (Eastern Sequencing and Informatics Hub, Cambridge, UK) and the reads of 36 nucleotides in length were aligned to the *T. brucei* TREU 927 genome v4 using Bowtie [15], [35]. Manipulation and processing of the aligned tags were done using SAMtools [36]. Aligned sequence tags were visualized using Artemis [37]. The number of hits per ORF was determined using HT-Seq software (http://www-huber.embl.de/users/anders/HTSeq/doc/index.html). Replicates were compared with the input RNA sample using the edgeR package [38]. The dataset was deposited in the European Nucleotide Archive (http://www.ebi.ac.uk/ena/data/view/PRJEB6768).

## Supporting Information

Figure S1 **Alignment of RBP33 from** ***T. brucei*** **,** ***T. cruzi*** **and** ***Leishmania major*** **.**
(PDF)

Figure S2 **Assesment of anti-RBP33 antiserum specificity and RBP33 abundance.** (A) A single immunoreactive band is visible in total cell extracts of bloodstream and procyclic trypanosomes in western blot assays. (B) RBP33 specific staining is no longer detected when the protein is ablated by RNAi in immunofluorescence analysis. (C) Quantitation of RBP33 protein levels in procyclic trypanosomes. The abundance of RBP33 present in different amounts of trypanosome cells (left) was compared to known amounts of recombinant His_6_-CBP-RBP33 [11] (right) in western blot assays.
(PDF)

Figure S3 **Examples of Artemis plots of RNA-seq data.** (A) Regions corresponding to genes *FTZC* (Tb927.10.8590), *RBP33* (Tb927.8.990) and Tb927.7.6010 are shown. (B) Transcripts derived from the SSR located in between genes Tb927.10.2390 and Tb927.10.2400 increase in abundance upon RBP33 depletion. Probes used for Northern hybridizations are indicated. Probe A is identical to that used in [19]. The annotated splicing acceptor site for Tb927.10.2390 is indicated with a filled circle, and the most proximal and most distal annotated polyadenylation sites are represented with empty circles.
(PDF)

Figure S4 **RBP33 depletion has no effect on the levels of** ***PT-X*** **and** ***FTZC*** **mRNAs or FTZC protein.** (A) Total RNA was obtained from trypanosomes grown in the presence or absence of tetracycline for 48 h, transferred to Nylon membranes and hybridized with probes designed to detect the *PT-X* and *FTZC* mRNAs. *7SL* RNA was used as a loading control. (B) Total cell extracts were obtained from parasites grown in the presence or absence of tetracycline for 48 h and subjected to western blot analysis using an antiserum against FTZC [41]. CSM protein was used as a loading control.
(PDF)

Figure S5 **RNaseH treatment.** To assess whether the transcripts detected with probe B in Figure 5 are polyadenyated, RNA samples obtained from RBP33-depleted cells were incubated with RNase H in the absence (−) or presence (+) of oligo(dT).
(PDF)

Table S1 **List of transcripts associated with RBP33.** Transcripts with a log_2_FC enrichment >3 (Sheet 1) or >2 (Sheet 2) with respect to the input sample are shown.
(XLSX)

Table S2 **Oligonucleotides used in this work.** List of the oligonucleotides designed for Northern and quantitative RT-PCR assays.
(XLSX)
