# Supplementary material for: Depletion of the RNA-Binding Protein RBP33 Results in Increased Expression of Silenced RNA Polymerase II Transcripts in Trypanosoma brucei
Source: PLoS One. 2014 Sep 12;9(9):e107608. doi: 10.1371/journal.pone.0107608 (PMC4162612; doi:10.1371/journal.pone.0107608)
Supplement: Figure S1 — Alignment of RBP33 from T. brucei , T. cruzi and Leishmania major . (PDF) [file pone.0107608.s001.pdf]

```
Tbrucei     1 MSTGDPTVPLEEEAISAEKCTRFFIGGLHRQITPQDVEEYFSNFGDVMTFILKRDPSGNS
Lmajor      1 ----------MPLTMHSSPHYKFFVSGIARHVSGDVVEAYFRGYGSGVRLQLMRDKDGLS
Tcruzi      1 MQVSDPTAASVEDTSSSVKDYRYFIGGIQRHITQQDIERYFSAFGEFEGFTMKRDSAGNS

Tbrucei    61 RGFGWVVFNSPPTGVQRPEPHYLKG--VKLTVEPALANV-----RTDRVRRVPGGGSSIP
Lmajor     51 LGYGWLTFESVDVERVVASQHELGGSPVMLRYQPGKKSPKSQPAASPPPPPPSQPINVPT
Tcruzi     61 RGYGWISYRSPPLGIARQDPHVLKG--VVLTVEQARSRGPHEGNRSEKRSYVNEGASLRR

Tbrucei   114 GRRRERSLSDSSSVSSRNSSLSTGDPRDGVRRPRLYDRHVEGSRLPAFGAVASRLPPLPS
Lmajor    111 QQGRS------QEYFPKHKRYREETFRPQVHTAS-RTAQIQTEIMPPKPQTLPGPQSLPK
Tcruzi    119 RRSRSPSSSSSSSVASRRGNYRR-------FTSSRQENANSTSRLPAIAVGNSRLPPLST

Tbrucei   174 EAFVQ-PPQIQATTFELAAPAPSR-DA--PAASASETYLCIPLSLCPPEFSNDPRTFCAK
Lmajor    164 ESRQP-ETEPSPTPMPQTVPLQHQFSSSPQEPVAQPVFACIPLSICPGAFLHDPRVFCCT
Tcruzi    172 QMYSSAPPAQSSTSTTIHGNVVST-NNCDDGNTVTETYLCIPITLCPSEYLSDPRTFCAR

Tbrucei   230 LDQSRVGGLSIIPSPSIAQVGPAPHSVPIPQGLP-GGTVMVPQHSRSQPVRSGGGGG---
Lmajor    223 LDP[illegible]QVGKLSILVTPTPHAALPNHHAEQAAYVVP-G--IPQQQHSTYASVNSKRVSPQTP
Tcruzi    231 LDQNRVGSLNIVPTPLVST-NAASVSVPPPPPPPPPSTRNLSQYGLIGERDSRNNAG---

Tbrucei   286 -GRHPKSSGSHHSIPPPLPQPAPPNYDSVMRHNS--------------------------
Lmajor    280 LSMPPLSSGPAPSPPPP-PGPPPPHLTAPFRETVPPPPGPPPPPPPPGPPPTKSCSSGIP
Tcruzi    287 -LPQSVSSSSSTTRVAMQRGMTH-------------------------------------

Tbrucei       --------------
Lmajor    339 LRFPPPPGPPPRRY
Tcruzi        --------------
```

RRM domain

Nuclear localization signal

Disordered
