# Supplementary figures and images for: Depletion of the RNA-Binding Protein RBP33 Results in Increased Expression of Silenced RNA Polymerase II Transcripts in Trypanosoma brucei
Source: PLoS One. 2014 Sep 12;9(9):e107608. doi: 10.1371/journal.pone.0107608 (PMC4162612; doi:10.1371/journal.pone.0107608)

**A**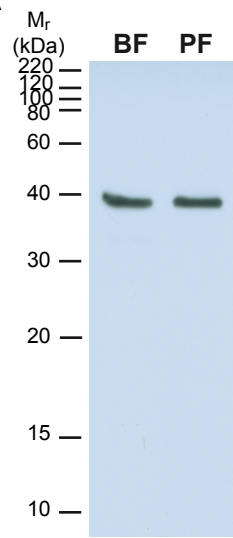**B**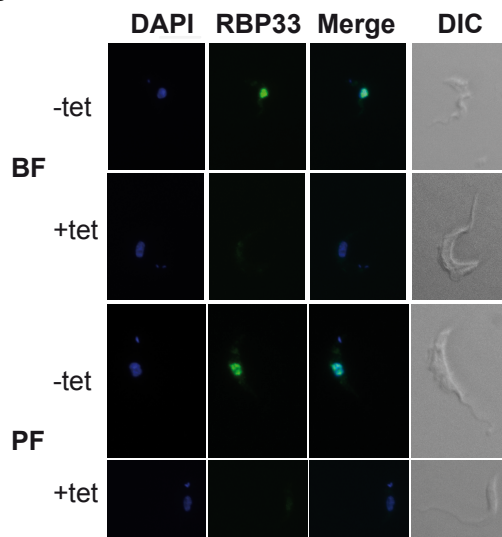**C**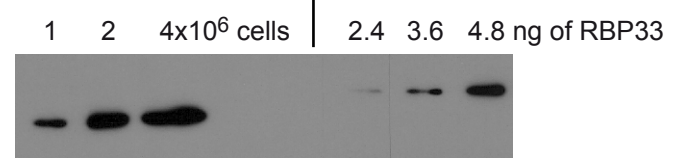

Supplement: Figure S2 — Assesment of anti-RBP33 antiserum specificity and RBP33 abundance. (A) A single immunoreactive band is visible in total cell extracts of bloodstream and procyclic trypanosomes in western blot assays. (B) RBP33 specific staining is no longer detected when the protein is ablated by RNAi in immunofluorescence analysis. (C) Quantitation of RBP33 protein levels in procyclic trypanosomes. The abundance of RBP33 present in different amounts of trypanosome cells (left) was compared to known amounts of recombinant His6-CBP-RBP33 [11] (right) in western blot assays. (PDF) [file pone.0107608.s002.pdf]

**A**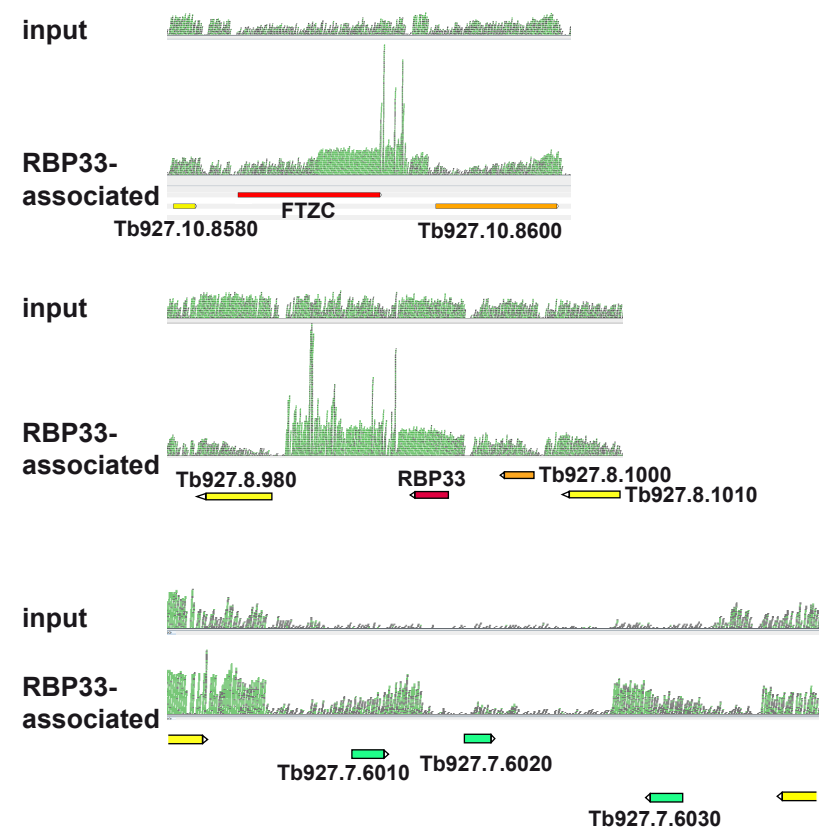**B**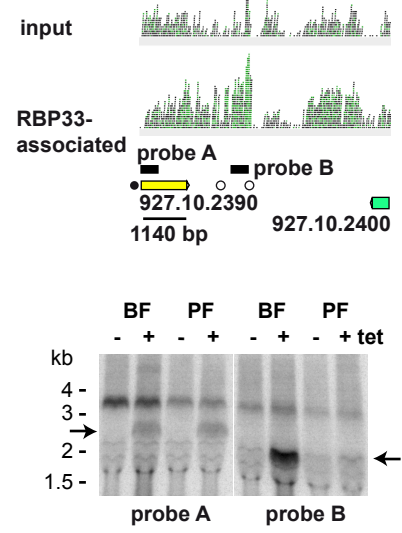

Supplement: Figure S3 — Examples of Artemis plots of RNA-seq data. (A) Regions corresponding to genes FTZC (Tb927.10.8590), RBP33 (Tb927.8.990) and Tb927.7.6010 are shown. (B) Transcripts derived from the SSR located in between genes Tb927.10.2390 and Tb927.10.2400 increase in abundance upon RBP33 depletion. Probes used for Northern hybridizations are indicated. Probe A is identical to that used in [19]. The annotated splicing acceptor site for Tb927.10.2390 is indicated with a filled circle, and the most proximal and most distal annotated polyadenylation sites are represented with empty circles. (PDF) [file pone.0107608.s003.pdf]

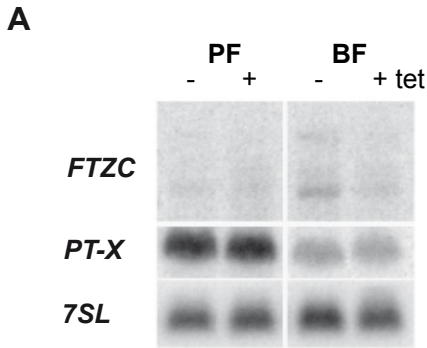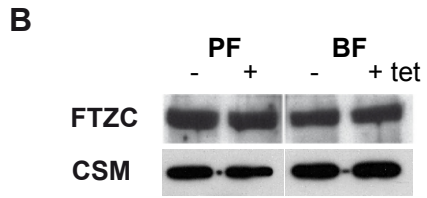

Supplement: Figure S4 — RBP33 depletion has no effect on the levels of PT-X and FTZC mRNAs or FTZC protein. (A) Total RNA was obtained from trypanosomes grown in the presence or absence of tetracycline for 48 h, transferred to Nylon membranes and hybridized with probes designed to detect the PT-X and FTZC mRNAs. 7SL RNA was used as a loading control. (B) Total cell extracts were obtained from parasites grown in the presence or absence of tetracycline for 48 h and subjected to western blot analysis using an antiserum against FTZC [41]. CSM protein was used as a loading control. (PDF) [file pone.0107608.s004.pdf]

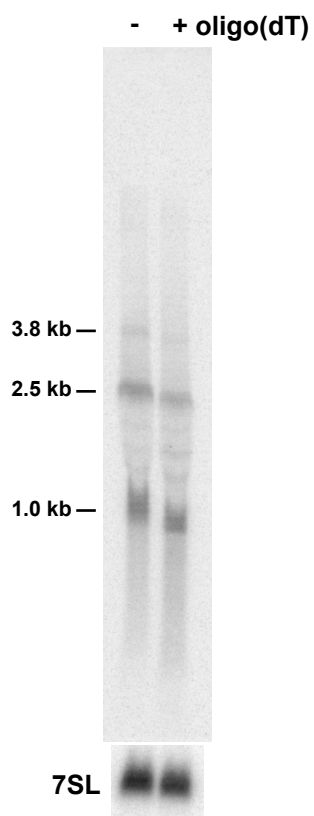

Supplement: Figure S5 — RNaseH treatment. To assess whether the transcripts detected with probe B in Figure 5 are polyadenyated, RNA samples obtained from RBP33-depleted cells were incubated with RNase H in the absence (−) or presence (+) of oligo(dT). (PDF) [file pone.0107608.s005.pdf]
